# Plasma endostatin may improve acute kidney injury risk prediction in critically ill patients

**DOI:** 10.1186/s13613-016-0108-x

**Published:** 2016-01-13

**Authors:** Johan Mårtensson, Niklas Jonsson, Neil J. Glassford, Max Bell, Claes-Roland Martling, Rinaldo Bellomo, Anders Larsson

**Affiliations:** Section of Anaesthesia and Intensive Care Medicine, Department of Physiology and Pharmacology, Karolinska Institutet, Solnavägen 1, 171 77 Solna, Sweden; Department of Intensive Care, Austin Hospital, 145 Studley Road, Heidelberg, VIC 3084 Australia; Australian and New Zealand Intensive Care Research Centre, School of Preventive Medicine and Public Health, Monash University, The Alfred Centre, 99 Commercial Road, Melbourne, VIC 3004 Australia; Department of Medical Sciences, Clinical Chemistry, Uppsala University, 751 85 Uppsala, Sweden

**Keywords:** Endostatin, Cystatin C, NGAL, Acute kidney injury, Sepsis

## Abstract

**Background:**

Breakdown of renal endothelial, tubular and glomerular matrix collagen plays a major role in acute kidney injury (AKI) development. Such collagen breakdown releases endostatin into the circulation. The aim of this study was to compare the AKI predictive value of plasma endostatin with two previously suggested biomarkers of AKI, cystatin C and neutrophil gelatinase-associated lipocalin (NGAL).

**Methods:**

We studied 93 patients without kidney disease who had a first plasma sample obtained within 48 h of ICU admission. We identified risk factors for AKI within the population and designed a predictive model. The individual ability and net contribution of endostatin, cystatin C and NGAL to predict AKI were evaluated by the area under the receiver operating characteristics curve (AUC), likelihood-ratio test, net reclassification improvement (NRI) and integrated discrimination improvement (IDI).

**Results:**

In total, 21 (23 %) patients experienced AKI within 72 h. A three-parameter model (age, illness severity score and early oliguria) predicted AKI with an AUC of 0.759 (95 % CI 0.646–0.872). Adding endostatin to the predictive model significantly (*P* = 0.04) improved the AUC to 0.839 (95 % CI 0.752–0.925). In addition, endostatin significantly improved risk prediction using the likelihood-ratio test (*P* = 0.005), NRI analysis (0.27; *P* = 0.04) and IDI analysis (0.07; *P* = 0.04). In contrast, adding cystatin C or NGAL to the three-parameter model did not improve risk prediction in any of the four analyses.

**Conclusions:**

In this cohort of critically ill patients, plasma endostatin improved AKI prediction based on clinical risk factors, while cystatin C and NGAL did not.

**Electronic supplementary material:**

The online version of this article (doi:10.1186/s13613-016-0108-x) contains supplementary material, which is available to authorized users.

## Background

Acute kidney injury (AKI) is common in the critically ill and associated with a high mortality rate [1]. Biomarkers may allow earlier detection of patients at risk of AKI and enable earlier intervention. Endostatin, the C-terminal fragment of collagen XVIII, is released into the circulation as a consequence of accelerated turnover of collagen XVIII in the basement membranes of the renal tubular epithelium, Bowman’s capsule, mesangium and renal capillaries, and may be one such biomarker [2].

In animal AKI models, upregulated renal endostatin expression preceded deteriorating kidney function by several hours [3, 4]. In addition, elevated serum endostatin has been associated with the degree of renal dysfunction in elderly patients and independently predicts the subsequent development of chronic kidney disease (CKD) in this population [5]. Endostatin has also been associated with mortality in several patient groups [6–8]. However, the potential of endostatin to identify critically ill patients at a higher risk of AKI remains unexplored.

Accordingly, we conducted a prospective, exploratory observational study to investigate the value of plasma endostatin as an early biomarker of AKI by comparing it with traditional clinical assessments of renal function and with plasma neutrophil gelatinase-associated lipocalin (NGAL) and plasma cystatin C. We hypothesized that, in patients with normal renal function on ICU admission, plasma endostatin levels would be higher among those patients who subsequently develop AKI compared with those who do not. In addition, we hypothesized that admission plasma endostatin would improve the AKI predictive ability of a clinical risk model.

## Methods

This study was approved by the regional ethical review board in Stockholm and has therefore been performed in accordance with the ethical standards laid down in the 1964 Declaration of Helsinki and its later amendments. Written informed consent was obtained from patients or their next of kin.

### Patient selection and operational definitions

We screened patients admitted to the general intensive care unit (ICU) at the Karolinska University Hospital, Solna, Sweden, from August 2007 to November 2010. We enrolled patients with an expected length of stay of more than 24 h and an estimated glomerular filtration rate (eGFR) of more than 60 mL/min/1.73 m^2^ (modification of diet in renal disease [MDRD] equation) on ICU admission.

We defined AKI as a ≥50 % increase in plasma creatinine from baseline or an increase in plasma creatinine by ≥26.5 µmol/L within 48 h and/or a urine output less than 0.5 mL/kg/h for at least 6 consecutive hours according to the Kidney Disease: Improving Global Outcomes (KDIGO) criteria [9]. We used the lowest creatinine level obtained within 3 months before ICU admission as baseline for the KDIGO classification. Missing baseline creatinine was imputed using the MDRD formula and an eGFR of 75 mL/min/1.73 m^2^ [9]. We decided a priori to exclude patients having their first study sample obtained >48 h after ICU admission and patients with AKI on the day of their first study sample collection. Our primary outcome was development of AKI within 72 h of first study sample collection. Accordingly, we recorded AKI status until a maximum of 5 days following ICU admission.

We defined the systemic inflammatory response syndrome (SIRS) using three or more SIRS criteria [10]. Sepsis was defined as a suspected or confirmed infection together with SIRS.

### Plasma sampling and biomarker analyses

We collected blood samples on study inclusion and twice daily thereafter until ICU discharge or start of renal replacement therapy. After centrifugation at 2000 rpm at 4 °C for 10 min, the supernatant plasma was stored at −80 °C.

Endostatin and NGAL were analyzed during 2013 using a commercially available enzyme-linked immunosorbent assay (ELISA) kit [DY1098 (endostatin) and DY1757 (NGAL), R&D Systems, Minneapolis, MN]. The assays had a total coefficient of variation (CV) of approximately 6 %. Cystatin C was measured with a particle-enhanced turbidimetric immunoassay on the Architect Ci8200 analyzer (Abbott Laboratories, Abbott Park, IL) with cystatin C reagents from Gentian (Moss, Norway).

### Statistical analysis

Data were analyzed using STATA^®^ version 11.2 software (Stata Corporation, College Station, TX, USA). Data are presented as medians and interquartile ranges (IQR) or as numbers and percentages. The Mann–Whitney test and Fisher’s exact test were used to test for differences between groups. The change over time for endostatin was tested by a repeated-measure analysis of variance (ANOVA). To compare the change over time between groups (AKI versus no AKI), we introduced an interaction variable between group and time to the ANOVA model. We assessed correlation using Spearman’s rank correlation coefficient (rho). The association of clinical variables with AKI development was assessed by multivariate logistic regression analysis. The following clinical predictor variables were considered: age, sex, acute physiology and chronic health evaluation (APACHE) II score, baseline creatinine, delta creatinine, early oliguria (urine output <0.5 mL/kg/h for >2 h but <6 h), presence of SIRS, presence of sepsis and noradrenaline dose. Clinical predictor variables were included in the multivariate models if they were statistically significant at *P* < 0.10 in the univariate analyses.

We assessed whether the addition of the measured biomarkers to the clinical model improved the predictive power for AKI by using the likelihood-ratio test. In addition, we calculated the area under the receiver operating characteristics curve (AUC) for the clinical model with and without inclusion of the measured biomarkers. The equality of AUCs was assessed by the method of DeLong et al. [11]. We described AUCs using the following values: 0.90–1.0 excellent, 0.80–0.89 good, 0.70–0.79 fair, 0.60–0.69 poor and 0.50–0.59 no useful performance [12]. The contributions of the biomarkers to risk prediction were further assessed by the net reclassification improvement (NRI) and the integrated discrimination improvement (IDI) methods. Log-transformed (base 10) biomarker values were used in the statistical analyses. Two-sided *P* values below 0.05 were considered statistically significant.

## Results

### Study patients

**Fig. 1:**
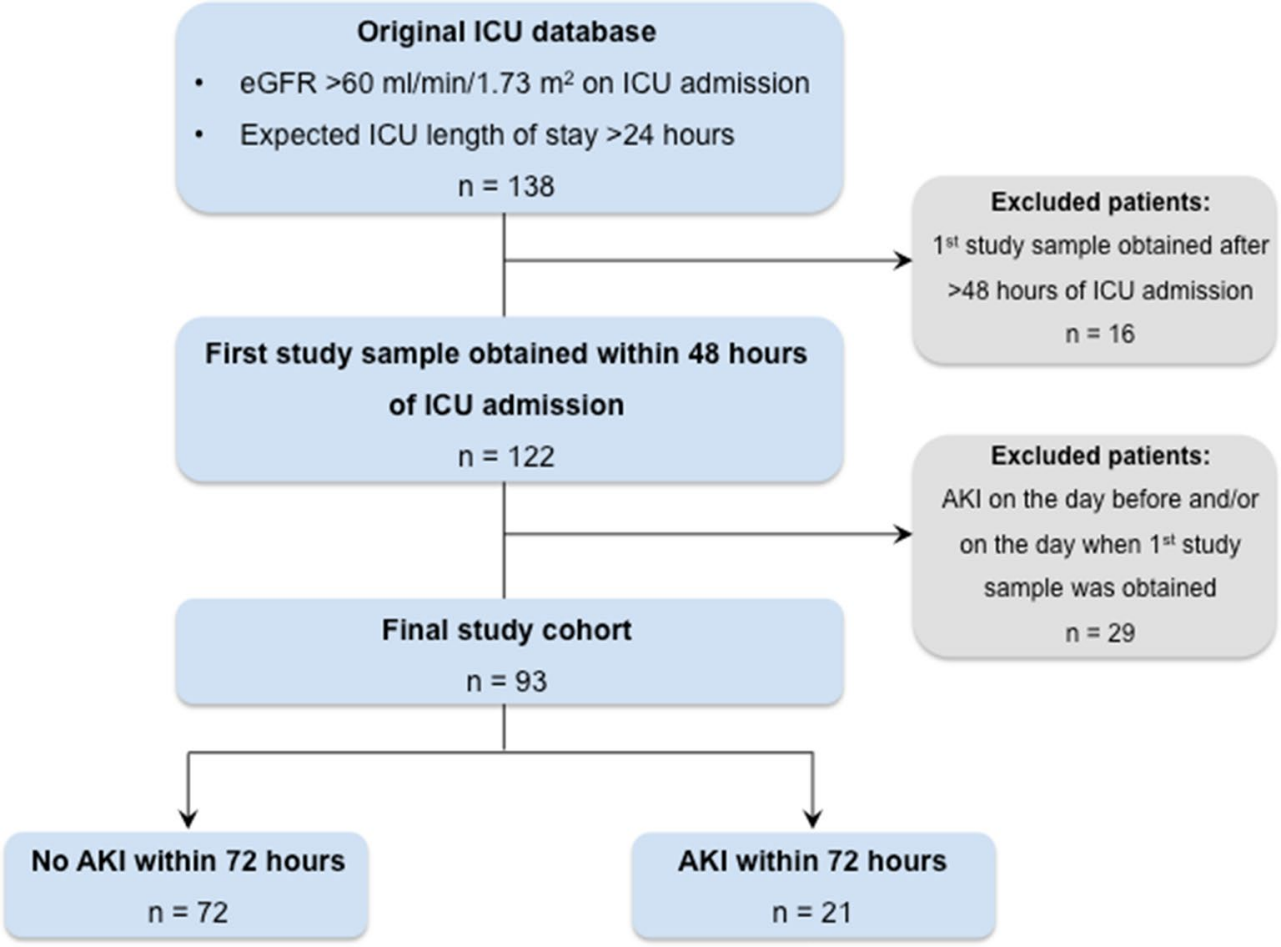
Selection of study patients

We enrolled 138 patients with eGFR > 60 mL/min/1.73 m^2^ and an expected length of stay >24 h (Fig. 1). We excluded 16 patients who were enrolled after >48 h of ICU admission and 29 patients with AKI on the day of their first study sample. We therefore studied 786 plasma samples in 93 patients; of these, 21 [22.6 % (95 % CI 14.6–32.4 %)] patients developed AKI within 72 h. Of the 21 AKI patients, 16 [76.2 % (52.8–91.8 %)] developed stage 1 AKI, 3 [14.3 % (3.0–36.3 %)] developed stage 2 AKI, 2 [9.5 % (1.2–30.4 %)] developed stage 3 AKI and 2 [9.5 % (1.2–30.4 %)] received renal replacement therapy.

Compared to non-AKI patients, AKI patients were older and had greater illness severity on presentation and more comorbidities (Table 1) but did not have significantly worse renal function at baseline. By 30 days after ICU admission, 4 (19 %) of 21 patients developing AKI had died compared to 8 (11 %) of the 72 non-AKI patients (*P* = 0.46).

**Table 1:** Patient characteristics and outcomes

Variable	No AKI (*n* = 72)	AKI (*n* = 21)	*P* value
Age (years)	50 (28, 65)	66 (57, 71)	0.002
Female gender	22 (31 %)	5 (24 %)	0.79
APACHE II score	15 (11, 19)	19 (14, 24)	0.01
Body weight (kg)	78 (70, 90)	88 (79, 93)	0.06
Baseline creatinine (µmol/l)	82 (69, 91)	83 (71, 88)	0.69
True baseline creatinine available	52 (72 %)	12 (57 %)	0.28
Time admission–enrolled (h)	12 (5, 23)	8 (3, 14)	0.10
Time from first biomarker analysis to AKI diagnosis (days)	N/A	1.0 (0.5, 1.5)	
Comorbidity			
Diabetes	6 (8 %)	5 (24 %)	0.12
Cardiovascular disease	20 (28 %)	11 (52 %)	0.06
COPD/asthma	5 (7 %)	2 (10 %)	0.65
Gastrointestinal/liver disease	2 (3 %)	3 (14 %)	0.07
Any malignancy	11 (15 %)	3 (14 %)	1.0
Admission diagnosis			
Neurologic	4 (6 %)	1 (5 %)	0.03
Respiratory	14 (19 %)	6 (29 %)	
Cardiovascular	2 (3 %)	5 (24 %)	
Trauma	37 (51 %)	7 (33 %)	
Gastrointestinal	3 (4 %)	1 (5 %)	
Sepsis	12 (17 %)	1 (5 %)	
Outcome			
ICU length of stay, days	5 (3, 9)	4 (3, 8)	0.72
ICU mortality	4 (6 %)	2 (10 %)	0.62
30-day mortality	8 (11 %)	4 (19 %)	0.46

Values are median (interquartile range) or *n* (%)

### Clinical renal characteristics at study inclusion

At inclusion, plasma creatinine levels (*P* = 0.20) and the changes in plasma creatinine from estimated or known baseline creatinine (*P* = 0.07) were similar (Table 2). Nine AKI patients (42.9 %) had early oliguria at inclusion, compared to 6 (8.3 %) non-AKI patients (*P* = 0.001). There was no difference in the prevalence of SIRS or sepsis at inclusion between groups (Table 2).

**Table 2:** Illness severity, organ function and inflammatory response at study inclusion

	No AKI (*n* = 72)	AKI (*n* = 21)	*P* value
Vasopressors and inotropes			
Noradrenaline dose (µg/kg/min)	0 (0, 0.03)	0.01 (0, 0.04)	0.43
Adrenaline [*n* (%)]	0 (0)	1 (4.8 %)	0.23
Dobutamine [*n* (%)]	1 (1.4 %)	3 (14.3 %)	0.04
Kidney function			
Plasma creatinine [µmol/l]	82 (69, 96)	91 (74, 93)	0.20
Δ creatinine [%]	0 (−12, 18)	8 (1, 25)	0.07
Early oliguria^a^ [*n* (%)]	6 (8.3 %)	9 (42.9 %)	0.001
Furosemide dose, mg during previous 24 h	6 (0, 23)	0 (0, 35)	0.93
Lung function			
Mechanical ventilation [*n* (%)]	56 (77.8 %)	16 (76.2 %)	1.0
FiO_2_	0.35 (0.26, 0.47)	0.40 (0.30, 0.50)	0.15
PaO_2_ (kPa)	10 (9, 11)	10 (9, 11)	0.95
PaO_2_/FiO_2_ ratio	29 (20, 42)	26 (18, 35)	0.31
PaCO_2_ (kPa)	4.8 (4.3, 5.4)	4.5 (4.3, 5.0)	0.12
Metabolic			
pH	7.4 (7.3, 7.4)	7.4 (7.3, 7.4)	0.64
Base excess (mmol/L)	0.2 (−2.4, 2.8)	−0.3 (−4.1, 1.0)	0.18
Lactate (mmol/L)	1.6 (1.2, 2.6)	2.1 (1.2, 2.8)	0.50
Inflammatory response			
SIRS [*n* (%)]	52 (72.2 %)	14 (66.7 %)	0.60
SIRS + suspected or confirmed infection [*n* (%)]	38 (52.8 %)	13 (61.9 %)	0.62
White cell count (×10^9^/L)	11 (8, 15)	10 (8, 13)	0.45
C-reactive protein (mg/dL)	74 (26, 181)	45 (15, 193)	0.56
Plasma biomarkers levels on inclusion			
Endostatin (ng/mL)	31 (23, 40)	42 (35, 54)	0.002
Cystatin C (mg/dL)	0.75 (0.64, 1.00)	1.10 (0.82, 1.40)	0.02
NGAL (ng/mL)	97 (66, 149)	133 (67, 180)	0.29

Values are reported as median (interquartile range) or as *n* (%)

^a^Urine output <0.5 mL/kg/h during >2 h but <6 h

### Biomarker characteristics at study inclusion

**Fig. 2:**
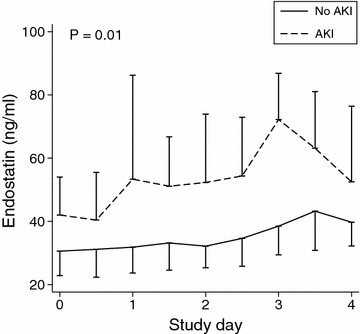
Plasma endostatin levels during the first five study days in AKI and non-AKI patients. Values are median and interquartile range. *P* value is for the repeated-measure ANOVA between groups

**Fig. 3:**
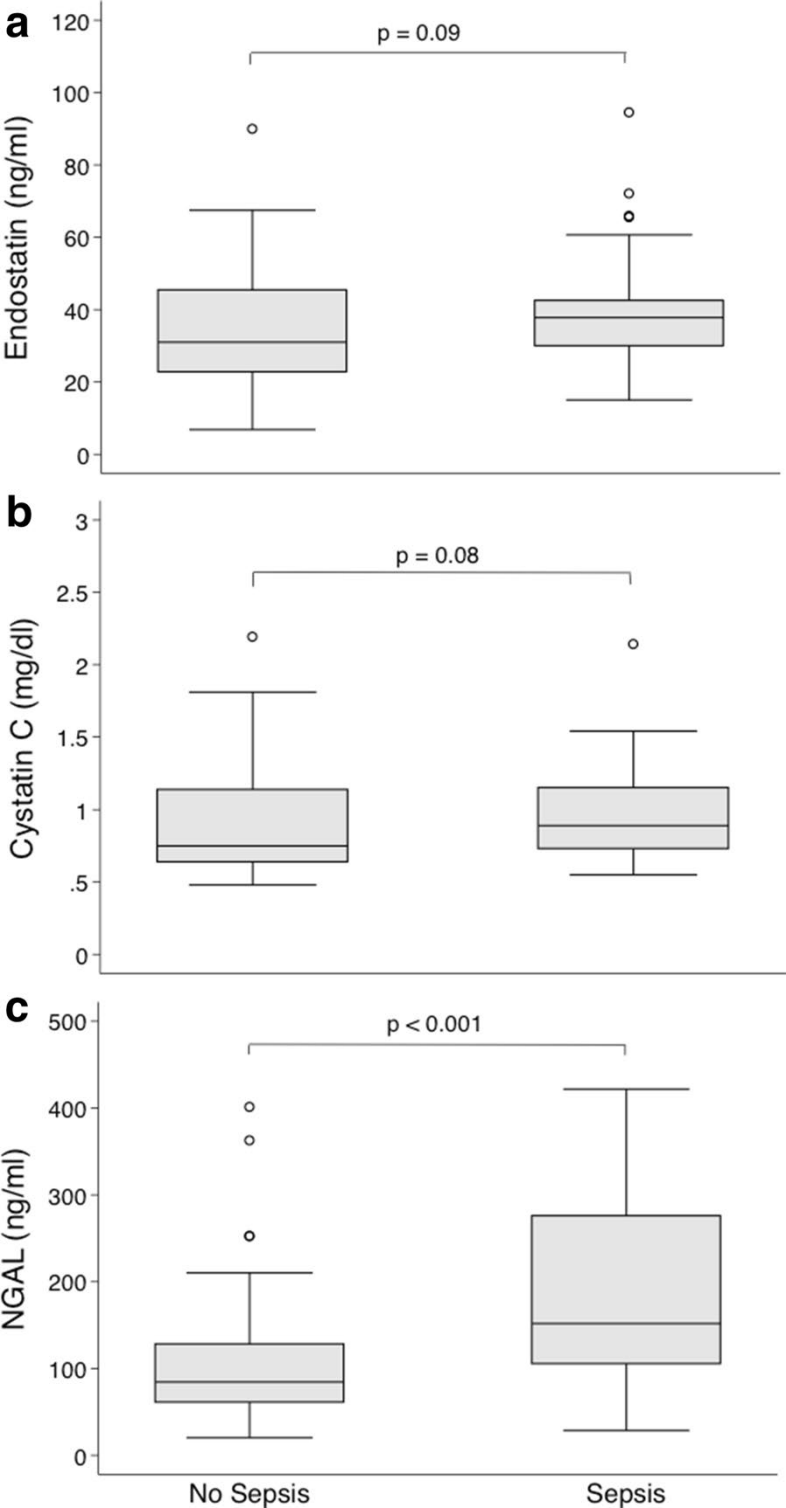
Plasma endostatin (**a**), cystatin C (**b**) and NGAL (**c**) at study inclusion in patients with and without sepsis

Plasma endostatin levels were significantly higher in patients who developed AKI (*P* = 0.002; Table 2) and remained higher during the first four study days (*P* = 0.01; Fig. 2). Inclusion cystatin C concentrations were also significantly greater in the AKI cohort at inclusion (*P* = 0.02; Table 2) and during the four study days (*P* = 0.002; Additional file 1: Fig. S1). However, we found no significant difference in plasma NGAL between the groups at inclusion (*P* = 0.29; Table 2) or over time (*P* = 0.06; Additional file 1: Fig. S2). Septic patients, as compared to non-septic patients, had higher plasma NGAL at inclusion (*P* < 0.001), whereas endostatin (*P* = 0.09) and cystatin C (*P* = 0.08) levels were similar in patients with and without sepsis (Fig. 3). We found a significant correlation between age and endostatin (Spearman’s rho 0.28, *P* = 0.006) and between age and cystatin C (Spearman’s rho 0.50, *P* < 0.001) but not between age and NGAL (Spearman’s rho 0.13, *P* = 0.17).

### Predicting the development of AKI

On univariate analysis, age, APACHE II score and early oliguria were associated with the development of AKI within 72 h and were included in a three-parameter clinical risk prediction model (Additional file 1: Table S1). This model predicted AKI with an AUC of 0.759 (95 % CI 0.646–0.872; Table 3).

**Table 3:** Values for prediction of AKI within 72 h

Statistic	Estimate (95 % CI)	*P* value^a^
AUC endostatin alone	0.726 (0.603 to 0.848)	<0.001
AUC cystatin C alone	0.674 (0.535 to 0.812)	0.01
AUC NGAL alone	0.577 (0.430 to 0.723)	0.22
AUC early oliguria	0.618 (0.509 to 0.727)	0.04
AUC clinical model	0.759 (0.646 to 0.872)	<0.001
AUC clinical model + endostatin	0.839 (0.752 to 0.925)	<0.001
AUC clinical model + cystatin C	0.776 (0.662 to 0.890)	<0.001
AUC clinical model + NGAL	0.766 (0.657 to 0.874)	<0.001
NRI (endostatin)	0.268 (0.010 to 0.526)	0.04
IDI (endostatin)	0.073 (0.038 to 0.142)	0.04
NRI (cystatin C)	0.06 (−0.16 to 0.28)	0.63
IDI (cystatin C)	0.012 (−0.013 to 0.037)	0.34
NRI (NGAL)	0.035 (−0.15 to 0.22)	0.71
IDI (NGAL)	0.0061 (−0.013 to 0.025)	0.53

*AUC* area under the receiver operating characteristics curve, *NRI* net reclassification improvement, *IDI* integrated discrimination improvement

^a^ *P* values for AUC assess the difference from 0.5

Endostatin alone predicted AKI with a similar AUC of 0.726 (0.603–0.848). Its optimal cutoff value of 37 ng/mL predicted AKI with a sensitivity of 71 % and a specificity of 65 % (Additional file 1: Table S2).

**Fig. 4:**
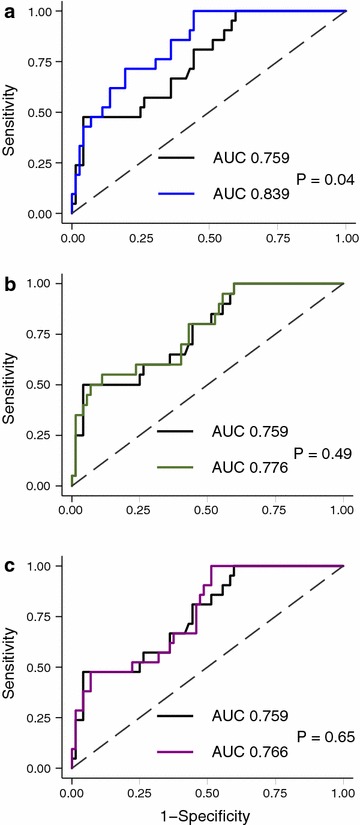
Receiver operating characteristics curves for prediction of AKI within 72 h using a clinical model (*black line*) and a clinical model together with endostatin (*blue line*,** a**), cystatin C (*green line*,** b**) or NGAL (*purple line*,** c**) at study inclusion. *AUC* area under the receiver operating characteristics curve

The addition of endostatin significantly improved the clinical three-parameter regression model, as confirmed by the likelihood-ratio test (*P* = 0.005; Additional file 1: Table S3). This endostatin-enhanced model was significantly better at predicting subsequent AKI than the clinical model alone, with an AUC of 0.839 (95 % CI 0.752–0.925; Table 3; Fig. 4), supported by both the net reclassification (*P* = 0.04; Table 3) and the integrated discrimination improvement indices (*P* = 0.04; Table 3). Cystatin C and NGAL were both poorly individually predictive of subsequent AKI (Table 3). Furthermore, the addition of either cystatin C or NGAL to the clinical predictive model failed to achieve any significant improvement in risk prediction (Table 3; Fig. 4; Additional file 1: Tables S4 and S5).

### Sensitivity analysis

We repeated the risk prediction analyses after removing age from the clinical model since age is a component of the APACHE II score. Both APACHE II and early oliguria were associated with AKI in this bivariate model. Adding endostatin to the bivariate model improved AKI prediction on the likelihood-ratio test (*P* < 0.001), the net reclassification improvement test (*P* = 0.02) and the integrated discrimination improvement test (*P* = 0.01). In contrast, adding cystatin C or NGAL did not improve risk prediction based on the bivariate model in any of the analyses (Additional file 1: Tables S6–S9 and Additional file 1: Fig. S3–S5).

## Discussion

### Key findings

We conducted a prospective, exploratory study assessing the relationship between a novel potential AKI biomarker (plasma endostatin) and AKI in critically ill patients. In this cohort, we found that plasma endostatin levels were higher in patients who developed AKI than in those who did not and that elevated plasma endostatin levels were independently associated with an increased risk of AKI. Moreover, the addition of plasma endostatin to a clinical prediction model significantly improved risk prediction performance for subsequent AKI. Finally, plasma endostatin concentration achieved greater utility in the prediction of subsequent AKI than either plasma NGAL or plasma cystatin C concentration.

### Relationship to previous studies

To our knowledge, this is the first investigation of plasma endostatin in critically ill humans as a predictor of AKI. However, in a cohort of elderly patients, serum endostatin levels independently predicted the future development of CKD [5]. In this population, endostatin also correlated strongly with eGFR. Moreover, up to fivefold higher endostatin levels have been observed in patients with CKD or ESRD [13, 14].

In the present study, inclusion endostatin levels were low and comparable to those observed in community-based cohorts (the PIVUS and ULSAM cohorts) [5]. Compared to these cohorts, our patients were markedly younger (median age 50–66 years versus a mean age >75 years in PIVUS and ULSAM) and did not have AKI or CKD on study inception. However, the majority of our critically ill patients had SIRS or sepsis at inclusion, which could be expected to affect endostatin levels in the same way it affects other biomarkers of AKI [15, 16]. For example, NGAL, which is released both by kidney epithelial cells and by activated neutrophils during systemic inflammation [17, 18], was elevated in our septic subgroup.

Similarly, collagen turnover is enhanced in sepsis, and this is reflected in elevated levels of circulating collagen degradation products [19]. Theoretically, matrix breakdown in non-renal tissues during sepsis could contribute to circulating endostatin levels. However, when comparing septic and non-septic patients we observed similar levels in both groups, suggesting minimal release of non-renal endostatin into the circulation in our patients.

Kidney extracellular matrix remodeling and angiogenesis appear to play an important role in the initiation, maintenance and progression of AKI [20, 21]. In experimental animal models of AKI, endothelial activation and damage lead to both disruption of renal endothelial cell integrity and endothelial matrix breakdown [3, 22]. This appears to be mediated via proteinase activation and cleavage of matrix collagens and leads to vascular leak, renal parenchymal edema and organ dysfunction [23]. Similar mechanisms have been demonstrated to contribute to the development of CKD [21]. Our finding that elevated circulating endostatin levels preceded AKI, as evident by a subsequent rise in creatinine, and remained elevated until such AKI was established supports the pathophysiological role of matrix breakdown and endothelial injury/dysfunction in AKI development. Additionally, we observed a slight increase in median endostatin levels in non-AKI patients reaching the optimal AKI predictive cutoff level (37 ng/mL) on day 3 (Fig. 2). Whether this delayed endostatin rise was triggered by some degree of “subclinical” AKI, by non-renal matrix breakdown or by both remains, however, uncertain.

Finally, as endostatin is a middle-sized molecule (20 kDa), which is freely filtered by the glomeruli, its plasma concentration may reflect glomerular filtration rate (GFR). In keeping with this hypothesis, an inverse correlation between plasma endostatin and GFR has indeed been established [5, 13]. Hence, impaired clearance of middle-sized molecules but not yet affecting smaller molecules such as creatinine (0.11 kDa) could potentially explain the observed early rise in endostatin. However, cystatin C (13 kDa) and NGAL (25 kDa) are both middle-sized molecules, but their patterns of release in our cohort differed from that of endostatin, and neither improved predictive modeling. In their aggregate, these observations suggest that increased production/release rather than impaired clearance of endostatin likely caused its early rise in plasma.

### Implications of study findings

Our findings may have implications for patient management. They support the emerging view that microcirculatory dysfunction is a key step in the establishment of AKI [24]. Endostatin may, in conjunction with other biomarkers of renal stress or injury, be a useful biomarker of the natural history of AKI and the transition from AKI to CKD. Within the limitations of this small cohort, our data suggest that elevated plasma endostatin may be an earlier and more specific signal of kidney stress or injury, especially in conjunction with clinical risk factors, than two previously proposed AKI markers: NGAL and cystatin C. Whether this signal is a useful tool to detect patients “at risk” of AKI, however, needs to be assessed in combination with validated clinical risk models in independent cohorts of critically ill patients. In particular, biomarker performance may be different in patients with sepsis. In fact, in a recent cohort of 112 septic patients, the ability to predict AKI within 24 h was fair using plasma cystatin C [AUC 0.737 (95 % CI 0.633–0.841)] and good using plasma NGAL [AUC 0.830 (95 % CI 0.741–0.919)] [25].

In contrast, cystatin C and NGAL demonstrated poor predictive performance in our study. This may be explained by the fact that we included both septic and non-septic patients and that we measured the occurrence of AKI up to 72 h after biomarker measurement. Based on these conflicting findings, future studies need to compare the AKI predictive performances of endostatin, cystatin C and NGAL in cohorts of septic patients and within different time-points of AKI diagnosis.

Promising therapies against acute renal microcirculatory changes have been demonstrated in animal AKI models but failed to improve kidney function or other patient-centered outcomes in clinical studies [24, 26]. Future studies should explore the role of plasma endostatin as a trigger of therapies targeting the renal microcirculation. Finally, endostatin may be particularly attractive in conjunction with novel imaging techniques of the renal microcirculation such as contrast-enhanced ultrasound [27, 28] and blood-oxygen level-dependent contrast imaging [29] to monitor therapeutic effects.

### Strengths and limitations

Our study has several strengths. It includes a well-defined cohort of critically ill patients without renal disease on ICU admission, yet at significant risk of developing AKI. Secondly, detailed data collection allowed us to develop a risk model using meaningful clinical and physiological parameters and to compare endostatin to established renal biomarkers like cystatin C and NGAL. Thirdly, data were prospectively collected and are therefore unlikely to be biased. Finally, despite a limited number of AKI cases, the added value of endostatin in risk prediction was consistently found in multiple analyses, which increases the robustness of our findings.

Our study has, however, limitations. It was a single-center study, which limits the generalizability of the study findings. It was, however, performed in a tertiary hospital suggesting some degree of external validity to similar hospitals in the developed world. CKD patients were excluded and the value of endostatin to predict acute-on-chronic kidney injury cannot be extrapolated from our findings. Illness severity (APACHE II) was generally low in our cohort. This was, however, expected since we excluded patients admitted with AKI, a population well known to be more severely ill. Furthermore, given our small study sample the robustness of our clinical risk model may be limited. However, our model includes clinically relevant and established risk factors (early oliguria, illness severity and age) of AKI. Additionally, we developed and validated the clinical risk model on the same cohort. This approach may inflate its predictive value. However, such potential inflation applied to all other biomarkers as well, and yet the addition of endostatin proved superior and significantly improved risk prediction. Finally, plasma samples were collected between 2007 and 2010 and analyzed for endostatin and NGAL during 2013. Such an extended storage period may impact biomarker concentrations. However, samples were stored at −80 °C, which preserve NGAL concentrations during long-term storage [30]. It is unlikely that identical storage conditions would affect endostatin levels differently.

## Conclusions

Our results suggest that plasma endostatin may be a fair predictor of AKI developing within 72 h of ICU admission in patients without preexisting renal disease. Combining endostatin with clinical variables may further enhance AKI risk prediction in such patients. In addition, within the limitations of an exploratory investigation, our findings suggest that endostatin may outperform two previously proposed AKI markers, cystatin C and NGAL. Our results provide the basis for further evaluation of endostatin as a biomarker of early AKI in independent cohorts of critically ill patients.

## Additional file

10.1186/s13613-016-0108-x **Tables S1–S5.** Clinical model and biomarker performances for AKI prediction. **Figures S1, S2.** Cystain C and NGAL kinetics. **Tables S6–S9 and Figures S3–S5.** Sensitivity analyses excluding age from clinical model.

## Abbreviations

AKI: acute kidney injury
APACHE: acute physiology and chronic health evaluation
AUC: area under the receiver operating characteristics curve
CKD: chronic kidney disease
ELISA: enzyme-linked immunosorbent assay
ESRD: end-stage renal disease
GFR: glomerular filtration rate
ICU: intensive care unit
IDI: integrated discrimination improvement
KDIGO: Kidney Disease: Improving Global Outcomes
MDRD: modification of diet in renal disease
NGAL: neutrophil gelatinase-associated lipocalin
NRI: net reclassification improvement
SIRS: systemic inflammatory response syndrome
