# Supplementary material for: Plasma endostatin may improve acute kidney injury risk prediction in critically ill patients
Source: Ann Intensive Care. 2016 Jan 13;6:6. doi: 10.1186/s13613-016-0108-x (PMC4712179; doi:10.1186/s13613-016-0108-x)
Supplement: Supplementary file 1 — 10.1186/s13613-016-0108-x Tables S1–S5. Clinical model and biomarker performances for AKI prediction. Figures S1, S2. Cystain C and NGAL kinetics. Tables S6–S9 and Figures S3–S5. Sensitivity analyses excluding age from clinical model. [file 13613_2016_108_MOESM1_ESM.docx]

**Supplementary Material**

**Plasma endostatin may improve acute kidney injury risk prediction in critically ill patients**

Johan Mårtensson, MD, PhD, Niklas Jonsson, MD, Neil J Glassford, MD, Max Bell, MD, PhD, Claes-Roland Martling, MD, PhD, Rinaldo Bellomo, MD, Anders Larsson, MD, PhD

**Table S1**. Univariate and multivariate logistic regression analysis assessing clinical predictors of AKI within 72 hours.

|  | **Univariate analysis** | | **Multivariate analysis** | |
| --- | --- | --- | --- | --- |
| **Predictor variables** | **Odds ratio**  **(95% CI)** | **P** | **Odds ratio**  **(95% CI)** | **P^a^** |
| Age,  per 1 year increase | 1.05 (1.02-1.08) | 0.003 | 1.04 (1.01-1.07) | 0.02 |
| Female sex | 0.71 (0.23-2.18) | 0.55 |  |  |
| APACHE II score,  per 1 score point increase | 1.10 (1.02-1.18) | 0.01 | 1.06 (0.98-1.15) | 0.14 |
| Baseline creatinine,  per 1 µmol increase | 0.99 (0.96-1.03) | 0.70 |  |  |
| Delta creatinine,  per 1 % increase | 1.02 (0.99-1.05) | 0.10 |  |  |
| Early oliguria^b^ | 4.64 (1.40-15.36) | 0.01 | 4.48 (1.18-17.06) | 0.03 |
| SIRS | 0.77 (0.27-2.18) | 0.62 |  |  |
| Sepsis | 1.30 (0.46-3.69) | 0.62 |  |  |
| Noradrenaline dose,  per 0.1 µg/kg/min increase | 1.34 (0.67-2.69) | 0.41 |  |  |
| ^a^Variables with a P<0.10 in the univariate analysis were included in the multivariate analysis  ^b^Urine output <0.5 ml/kg/h during >2 hours but <6 hours | | | | |

**Table S2**. Value of endostatin, cystatin C and NGAL to predict AKI within 72 hours.

| **Biomarker** | **AUC ROC** | **Cut-off** | **Sensitivity** | **Specificity** |
| --- | --- | --- | --- | --- |
| Endostatin | 0.73 (0.60-0.85) | 37 ng/ml | 0.71 (0.48-0.89) | 0.65 (0.53-0.76) |
| Cystatin C | 0.67 (0.54-0.81) | 1.1 mg/l | 0.55 (0.32-0.77) | 0.76 (0.65-0.86) |
| NGAL | 0.58 (0.43-0.72) | 150 ng/ml | 0.38 (0.18-0.62) | 0.76 (0.65-0.86) |

**Table S3**. Clinical risk model and clinical + endostatin risk model for AKI.

|  | **Clinical Model^a^** | | **Clinical Model + Endostatin** | |
| --- | --- | --- | --- | --- |
| **Predictor variables** | **Odds Ratio**  **(95% CI)** | ***P* value** | **Odds Ratio**  **(95% CI)** | ***P* value^d^** |
| Age,  per 1-year increase | 1.04 (1.01-1.07) | 0.02 | 1.03 (0.99-1.08) | 0.10 |
| APACHE II score,  per 1 score point increase | 1.06 (0.98-1.15) | 0.14 | 1.09 (1.00-1.19) | 0.06 |
| Early oliguria^c^ | 4.48 (1.18-17.06) | 0.03 | 4.63 (1.08-19.8) | 0.04 |
| Endostatin^b^,  per 0.1 log_10_-unit increase | Not included in model | | 1.56 (1.12-2.19) | 0.01 |
| ^a^Variables with a P<0.10 on univariate analysis were included in the Clinical Model  ^b^Endostatin was log 10 transformed (base 10).  ^c^Urine output <0.5 ml/kg/h during >2 hours but <6 hours  ^d^Addition of endostatin improved the clinical model significantly (P = 0.005 on likelihood-ratio test) | | | | |

**Table S4**. Clinical risk model and clinical + cystatin C risk model for AKI.

|  | **Clinical Model^a^** | | **Clinical Model + Cystatin C** | |
| --- | --- | --- | --- | --- |
| **Predictor variables** | **Odds Ratio**  **(95% CI)** | ***P* value** | **Odds Ratio**  **(95% CI)** | ***P* value** |
| Age,  per 1-year increase | 1.04 (1.01-1.07) | 0.02 | 1.03 (0.99-1.06) | 0.14 |
| APACHE II score,  per 1 score point increase | 1.06 (0.98-1.15) | 0.14 | 1.08 (0.99-1.17) | 0.09 |
| Early oliguria^b^ | 4.48 (1.18-17.06) | 0.03 | 4.84 (1.25-18.72) | 0.02 |
| Cystatin C^c^,  per 1.0 log_10_-unit increase | Not included in model | | 6.54 (0.14-312.84) | 0.52 |
| ^a^Variables with a P<0.10 on univariate analysis were included in the Clinical Model  ^b^Urine output <0.5 ml/kg/h during >2 hours but <6 hours  ^c^Cystatin C was log 10 transformed | | | | |

**Table S5**. Clinical risk model and clinical + NGAL risk model for AKI.

|  | **Clinical Model^a^** | | **Clinical Model + NGAL** | |
| --- | --- | --- | --- | --- |
| **Predictor variables** | **Odds Ratio**  **(95% CI)** | ***P* value** | **Odds Ratio**  **(95% CI)** | ***P* value** |
| Age,  per 1-year increase | 1.04 (1.01-1.07) | 0.02 | 1.04 (1.00-1.07) | 0.03 |
| APACHE II score,  per 1 score point increase | 1.06 (0.98-1.15) | 0.14 | 1.06 (0.98-1.16) | 0.13 |
| Early oliguria^b^ | 4.48 (1.18-17.06) | 0.03 | 4.46 (1.15-17.27) | 0.03 |
| NGAL^c^,  per 1.0 log_10_-unit increase | Not included in model | | 2.14 (0.34-13.58) | 0.42 |
| ^a^Variables with a P<0.10 on univariate analysis were included in the Clinical Model  ^b^Urine output <0.5 ml/kg/h during >2 hours but <6 hours  ^c^NGAL was log 10 transformed | | | | |

**Figure S1**. Plasma cystatin C levels during the first five study days in AKI and non-AKI patients. Values are median and interquartile range. P-value is for the repeated-measure ANOVA between groups.

**Figure S2**. Plasma NGAL levels during the first five study days in AKI and non-AKI patients. Values are median and interquartile range. P-value is for the repeated-measure ANOVA between groups.

**Sensitivity Analyses excluding AGE from clinical model**

**Table S6**. Bivariate clinical risk model (without age) + endostatin risk model for AKI.

|  | **Clinical Model^a^** | | **Clinical Model + Endostatin** | |
| --- | --- | --- | --- | --- |
| **Predictor variables** | **Odds Ratio**  **(95% CI)** | ***P* value** | **Odds Ratio**  **(95% CI)** | ***P* value^d^** |
| APACHE II score,  per 1 score point increase | 1.10 (1.02-1.18) | 0.02 | 1.12 (1.03-1.22) | 0.009 |
| Early oliguria^c^ | 4.39 (1.26-15.23) | 0.02 | 4.56 (1.12-18.51) | 0.03 |
| Endostatin^b^,  per 0.1 log_10_-unit increase | Not included in model | | 1.63 (1.19-2.25) | 0.003 |
| ^a^Variables with a P<0.10 on univariate analysis were included in the Clinical Model  ^b^Endostatin was log 10 transformed (base 10).  ^c^Urine output <0.5 ml/kg/h during >2 hours but <6 hours  ^d^Addition of endostatin improved the clinical model significantly (P<0.001on likelihood-ratio test) | | | | |

**Figure S3**. Receiver operating characteristics curves for prediction of AKI within 72 hours using a clinical model (open circles) and a clinical model together with endostatin (closed circles). *AUC* area under the receiver operating characteristics curve.

**Table S7**. Clinical risk model (without age) + cystatin C risk model for AKI.

|  | **Clinical Model^a^** | | **Clinical Model + Cystatin C** | |
| --- | --- | --- | --- | --- |
| **Predictor variables** | **Odds Ratio**  **(95% CI)** | ***P* value** | **Odds Ratio**  **(95% CI)** | ***P* value^d^** |
| APACHE II score,  per 1 score point increase | 1.10 (1.02-1.18) | 0.02 | 1.10 (1.01-1.19) | 0.02 |
| Early oliguria^b^ | 4.39 (1.26-15.23) | 0.02 | 4.78 (1.29-17.66) | 0.02 |
| Cystatin C^c^,  per 1.0 log_10_-unit increase | Not included in model | | 25.62 (0.80-816) | 0.07 |
| ^a^Variables with a P<0.10 on univariate analysis were included in the Clinical Model  ^b^Urine output <0.5 ml/kg/h during >2 hours but <6 hours  ^c^Cystatin C was log 10 transformed  ^d^Addition of cystatin C did not improve the clinical model significantly (P=0.06 on likelihood-ratio test) | | | | |

**Figure S4**. Receiver operating characteristics curves for prediction of AKI within 72 hours using a clinical model (open circles) and a clinical model together with cystatin C (closed circles). *AUC* area under the receiver operating characteristics curve.

**Table S8**. Clinical risk model (without age) + NGAL risk model for AKI.

|  | **Clinical Model^a^** | | **Clinical Model + NGAL** | |
| --- | --- | --- | --- | --- |
| **Predictor variables** | **Odds Ratio**  **(95% CI)** | ***P* value** | **Odds Ratio**  **(95% CI)** | ***P* value^d^** |
| APACHE II score,  per 1 score point increase | 1.06 (0.98-1.15) | 0.14 | 1.10 (1.02-1.18) | 0.02 |
| Early oliguria^b^ | 4.48 (1.18-17.06) | 0.03 | 4.42 (1.24-15.72) | 0.02 |
| NGAL^c^,  per 1.0 log_10_-unit increase | Not included in model | | 2.98 (0.46-19.23) | 0.25 |
| ^a^Variables with a P<0.10 on univariate analysis were included in the Clinical Model  ^b^Urine output <0.5 ml/kg/h during >2 hours but <6 hours  ^c^NGAL was log 10 transformed  ^d^Addition of NGAL did not improve the clinical model significantly (P=0.24 on likelihood-ratio test) | | | | |

**Figure S5**. Receiver operating characteristics curves for prediction of AKI within 72 hours using a clinical model (open circles) and a clinical model together with NGAL (closed circles). *AUC* area under the receiver operating characteristics curve.

**Table S9**. Values for prediction of AKI within 72 hours. Age excluded from clinical model.

| **Statistic** | **Estimate**  **(95% CI)** | **P value^a^** |
| --- | --- | --- |
| AUC endostatin alone | 0.726 (0.603-0.848) | <0.001 |
| AUC cystatin C alone | 0.674 (0.535-0.812) | 0.01 |
| AUC NGAL alone | 0.577 (0.430-0.723) | 0.22 |
| AUC clinical model | 0.729 (0.596-0.862) | <0.001 |
| AUC clinical model + endostatin | 0.831 (0.741-0.922) | <0.001 |
| AUC clinical model + cystatin C | 0.757 (0.630-0.884) | <0.001 |
| AUC clinical model + NGAL | 0.724 (0.588-0.860) | <0.001 |
| NRI (endostatin) | 0.385 (0.072-0.698) | 0.02 |
| IDI (endostatin) | 0.101 (0.024-0.177) | 0.01 |
| NRI (cystatin C) | -0.072 (-0.355-0.211) | 0.62 |
| IDI (cystatin C) | 0.046 (-0.001-0.093) | 0.06 |
| NRI (NGAL) | -0.165 (-0.382-0.053) | 0.14 |
| IDI (NGAL) | 0.018 (-0.045-0.010) | 0.21 |
| *AUC* area under the receiver operating characteristics curve, *NRI* net reclassification improvement, *IDI* integrated discrimination improvement  ^a^P-values for AUC assess the difference from 0.5 | | |
